# Effectiveness of an interprofessional education program using team‐based learning for medical students: A randomized controlled trial

**DOI:** 10.1002/jgf2.284

**Published:** 2019-11-03

**Authors:** Shuhei Hamada, Junji Haruta, Takami Maeno, Tetsuhiro Maeno, Hideo Suzuki, Ayumi Takayashiki, Haruhiko Inada, Takahiro Naito, Mika Tomita, Naomi Kanou, Takeshi Baba

**Affiliations:** ^1^ Graduate School of Comprehensive Human Sciences University of Tsukuba Tsukuba Japan; ^2^ Department of Primary Care and Medical Education Faculty of Medicine University of Tsukuba Tsukuba Japan; ^3^ Faculty of Medicine University of Tsukuba Tsukuba Japan; ^4^ Johns Hopkins International Injury Research Unit Department of International Health Johns Hopkins Bloomberg School of Public Health Baltimore MD USA; ^5^ Center for Fundamental Education Teikyo University of Science Tokyo Japan; ^6^ Department of Nursing School of Health Sciences Ibaraki Prefectural University of Health Sciences Ami Japan; ^7^ Center for Medical Sciences Ibaraki Prefectural University of Health Sciences Ami Japan

**Keywords:** medical education, research

## Abstract

**Background:**

To respond to increasingly complicated healthcare needs in primary care settings, all health and medical welfare professionals are required to collaborate with multiprofessionals, namely via “interprofessional work” (IPW). Interprofessional education (IPE) is essential for effective IPW, especially for medical students. This study aimed to determine whether participation in IPE can increase medical students’ readiness for interprofessional learning.

**Method:**

We examined the difference in readiness of medical students for interprofessional learning before and after an IPE program that used team‐based learning (TBL). Each student was assigned to either a uniprofessional or multiprofessional group. They were evaluated using the Japanese version of the Readiness for Interprofessional Learning Scale (RIPLS). Program participants were 126 second‐year medical students and 18 students of healthcare professions other than medical doctor who participated in a combined IPE program conducted by two universities. Medical students were allocated to 12 uniprofessional and nine multiprofessional groups at random.

**Results:**

One hundred and twelve medical students who replied to the questionnaire both before and after the program (valid response rate, 88.9%) were eligible for analysis. Of these, 42 were assigned to uniprofessional groups and 70 to multiprofessional groups. After the program, the RIPLS total score increased to a greater extent in the multiprofessional groups than in the uniprofessional groups (difference 3.17, 95% confidence interval 0.47‐5.88, *P* = .022). Multiple regression analysis showed the same result.

**Conclusions:**

Learning in multiprofessional groups increased medical students’ readiness for interprofessional learning in an IPE program using TBL.

## Graphical abstract

In our Interprofessional edcation (IPE) program that used a team‐based learning (TBL) format for second‐year medical students, medical students who were assigned to multi‐professional groups had improved readiness towards interprofessional learning compared with those in uni‐professional groups. IPE using TBL may be helpful to improve readiness towards future onterprofessional work.

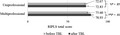

## 1 INTRODUCTION

To respond to increasingly complicated healthcare needs in primary care settings, all health and medical welfare professionals are required to cooperate and collaborate across disciplines, a practice referred to as “interprofessional work” (IPW).^1^ Solving complicated care problems and providing the necessary services require teamwork in IPW as well as sharing a team identity.^2^

However, doctors who often collaborate with other medical professionals in the context of medical care provision tend have a negative attitude toward IPW.^3^, ^4^, ^5^ Physicians with a negative attitude toward IPW often believe that the role of doctors is to make a decision in a top‐down approach within medical teams.^3^, ^6^ Such thinking may pose a barrier to IPW in the field. To overcome this situation, WHO has indicated the need to implement interprofessional education (IPE) to improve the preparation of health and medical welfare professionals to collaborate in multidisciplinary healthcare settings.^7^ IPE is defined as “occasions when two or more professions learn with, from and about each other to improve collaboration and the quality of care”.^8^ Some studies targeting medical students have reported that students who have received IPE tend to have a positive attitude toward IPW.^9^, ^10^ Through IPE, medical students can practice the doctor's role and experience interactions with students of other professions.^8^

In Europe and the United States, efforts to implement IPE began about 20 years ago, and the number of universities that incorporate IPE into their curriculum is rapidly increasing.^11^ Positive effects have been reported, including improved knowledge,^12^ improved preparation for interprofessional collaboration,^13^ and increased satisfaction toward IPE learning after involvement in IPE programs,^14^ such as small group learning and practical training in a group of multiprofessionals. In particular, because recent overcrowding of the medical curricula has hampered efforts to insert IPE programs into the overall curriculum,^15^ a more effective multiprofessional collaborative education program is needed to improve the readiness of students for multiprofessional collaboration in a limited period of time. While some studies have reported that learning in a multiprofessional group composed of students from different professions, as opposed to a uniprofessional group, improves readiness for interprofessional learning,^16^, ^17^ the analyses were often conducted without distinguishing between different professions.

Further, reports limited to analyzing the readiness of medical students only for collaboration in healthcare environments are scarce. In a study of clinical practical training for the care of children with disabilities in which medical students were randomly paired with either medical students or nursing students, those paired with nursing students showed no significant improvement in attitude toward IPE compared with those paired with medical students.^17^ One study assigned medical students to either a uniprofessional group of medical students or a multiprofessional group of pharmacy and nursing students, and evidence‐based medical learning based on a patient scenario was conducted in a small group. The results indicated that the medical students who were assigned to the multiprofessional group showed improved clinical decision‐making ability compared with those assigned to the uniprofessional group.^18^ As the outcome was clinical decision‐making ability, the study did not evaluate whether placement in a multiprofessional group improved the medical students’ readiness for interprofessional collaboration. In educational research, while it is possible to more accurately measure the educational effect by random assignment, random assignment at actual educational sites is difficult. Apart from the two papers mentioned above, few reports have focused on the random assignment of medical students to uniprofessional or multiprofessional groups and compared the effects of these assignments on their education.^17^, ^18^

In this study, we randomly assigned medical students to uniprofessional or multiprofessional groups in an IPE program. We then studied whether learning in a multiprofessional group improved medical students’ readiness for interprofessional learning compared to learning in a uniprofessional group composed of medical students only.

## 2 METHODS

### 2.1 Overview of implementation of the IPE program

Program participants were second‐year medical students of X University and second‐year students of nursing, physical therapy, occupational therapy, and radiological technology at Y University who were taking part in a combined IPE program conducted by the two universities in October 2013. The purpose of the program was for students to learn the importance of team medicine and professional cooperation. This program was a compulsory subject at X University, School of Medicine, and 126 medical students participated, while it was an elective course at Y University, involving four nursing, eight physical therapy, five occupational therapy, and one radiology student. The Team‐Based Learning (TBL)^19^ format was used as the learning strategy. We developed a TBL course based on a case of cerebral infarction in which the patient was aiming to return home after recovery from the acute stage. We chose rehabilitation from cerebral infarction because multiple professions are involved in its treatment and care. All instructors involved in the students’ curriculum participated in faculty development of TBL and IPE. Faculty members from Y University were nursing, physical therapy, occupational therapy, and radiology instructors. A small group (about six or seven people in one group) occupied two large classrooms, and 7‐8 instructors comprising one or more medical, nursing, physical therapy, occupational therapy, and radiology faculty members were present in each classroom. The faculty members’ role was primarily to watch over the students’ progress in learning, and they were not in charge of a specific group. They were allowed to provide advice when the students became stuck in discussion, although this did not occur in any group. Participating students were grouped in advance. Because the total number of nursing, physical therapy, occupational therapy, and radiology students was lower than that of medical students, it was not possible for all students to join a multiprofessional group. Medical students were randomly assigned to uniprofessional groups of medical students only or multiprofessional groups using a random number table. All nursing, physical therapy, occupational therapy, and radiology students were allotted to multiprofessional groups. Program participants were divided into 12 uniprofessional groups (6‐7 medical students from X University in one group) and nine multiprofessional groups (five medical students from X University + two students from Y University in one group). As a result, 81 medical students were assigned to uniprofessional groups and 45 to multiprofessional groups. In the multiprofessional groups, there was no duplication of nursing, physical therapy, occupational therapy, and radiology students.

The medical students had undergone TBL one year before. Seven days before the scheduled group learning session, participants received an explanation of the implementation of TBL as a learning task to raise their level of individual readiness (preclass self‐learning). After the preliminary explanation and before TBL implementation, we posted the group assignments. On the day of TBL, a half‐day program was implemented. After orientation and icebreaking, we conducted the individual readiness assurance test (IRAT), group readiness assurance test (GRAT), and faculty feedback and clarification (mini‐lecture) based on the learning task for readiness assurance. Subsequently, the faculty members introduced the mock case on the acute and rehabilitative stages of cerebral infarction and encouraged the program participants to discuss the types of problems embedded in the case and strategies to solve them. Faculty members of X University Medical Institute and Y University facilitated the discussion in each classroom. They also commented on students’ presentations, which were attended by all students in both groups.

### 2.2 Study design

Randomized controlled study.

### 2.3 Study participants

Eligible study participants were 126 medical students who participated in the IPE program on October 31, 2013. Participants had not yet taken the Common Achievement Tests (a Japanese nationwide examination similar to the USMLE Step 1 examination).

### 2.4 Research method

At the start and end of the IPE program, participants were asked to complete the Japanese version of the Readiness for Interprofessional Learning Scale (RIPLS)^20^, ^21^ and their responses were compiled to evaluate the program. We assured the participants that their answers would not influence their grades. The RIPLS was conducted immediately before the program orientation and after the program had concluded. The questionnaires were collected on the spot.

### 2.5 Questionnaire: Japanese version of RIPLS^21^

Readiness for Interprofessional Learning Scale^20^ was developed as a self‐administered questionnaire for assessing health professional students’ readiness for interprofessional learning in IPE. It is considered a valid and reliable tool and has been translated into several languages.^22^, ^23^ Tamura et al^21^ evaluated the validity and reliability of the Japanese version of the RIPLS in 2010. The Japanese version of RIPLS contains 19 items categorized under three subscales: “team and collaborations” (13 items), “IPE opportunity” (two items), and “uniqueness of profession” (four items). The items are measured on a 5‐point Likert scale with responses ranging from “strongly agree” to “strongly disagree.” The RIPLS score, which includes five reverse‐scored items, indicates the readiness of professionals for IPE. Higher RIPLS total scores in this study therefore indicate greater readiness of undergraduate healthcare students for interprofessional learning. Studies have mainly examined the effect of IPE by evaluating the results of the whole questionnaire. We evaluated students’ readiness for IPE by examining the mean difference in the RIPLS total score before and after the IPE program.

### 2.6 Statistical analysis

Each item was rated on a 5‐point Likert scale from “strongly agree” to “strongly disagree” in the Japanese version of RIPLS. Each value on the scale was assigned points (from 5 to 1) and used for analysis. Negatively worded items were reverse‐scored. We analyzed the mean difference in the RIPLS total score before and after the TBL program using the paired *t* test separately in the uniprofessional and multiprofessional groups. Moreover, to examine the change in attitude of medical students toward IPE, we analyzed the difference in RIPLS total score before and after the TBL program between uniprofessional and multiprofessional groups using the unpaired Student's *t* test. We used multiple regression analysis (forced entry method) to adjust for the effect of potential confounders. The regression model included the mean difference in RIPLS total score before and after the TBL program as a dependent variable, and RIPLS total score before the TBL program, group (multi‐/uniprofessional), age, and gender as independent variables. Categorical data such as group, age, and gender were coded as dummy variables. A *P* value of <0.05 was considered statistically significant. Statistical analysis was performed using SPSS Statistics 22 (IBM Japan).

### 2.7 Sample size

We assumed that the mean total difference in RIPLS (19 items: 95 points, full marks) score (standard variation) before and after the IPE program was 6.0 (5.0) points based on the results of our previous study using RIPLS to assess IPE in our university. The difference was considered significant when the mean total difference in RIPLS score before and after the program in the uni‐/multiprofessional groups was greater than 60%, the effect size was 6.0 × 0.6 = 3.6 points, and the standardized effect size was 3.6/5.0 = 0.72. The required sample size was calculated to be 34 in each group with a significance level of (two‐sided) *α* = 0.05 and statistical power 1‐*β* = 0.80. All students who were planning to participate in the IPE program were targeted in this study.

### 2.8 Ethical considerations

We explained to the participants that the results of the RIPLS would be used to evaluate the program and obtained consent from the students for the use of their results in this study. This study was approved by the authors' institutional ethics committee.

## 3 RESULTS

### 3.1 Sample and characteristics

**Figure 1:**
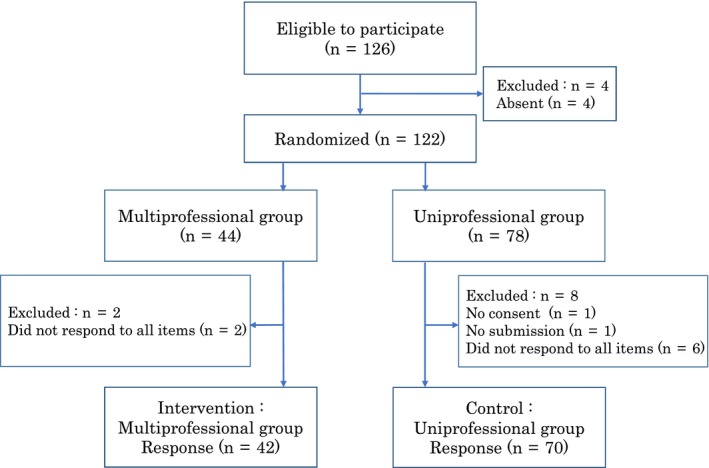
The flow of participants in this study

Figure 1 shows the flow of participants in this study (Figure 1). A total of 112 students were analyzed: 70 in the uniprofessional group and 42 in the multiprofessional group (valid response rate, 88.9%). Baseline characteristics (gender and age) were as follows: There were 49 males (70.0%) and 21 females (30.0%) in the uniprofessional group with a mean age of 20.4 (± 1.6) years, and 29 males (69.0%) and 13 females (31.0%) in the multiprofessional group with a mean age of 21.1 (± 3.4) years. There were no statistical differences in baseline characteristics between the groups (Table 1).

**Table 1:** Characteristics of the study population

Characteristic	Total (n = 112)		
	Uniprofessional group (n = 70)	Multiprofessional group (n = 42)	*P* value
Age, mean ± SD	20.4 ± 1.6	21.1 ± 3.4	.20^a^
Gender			
Male, n (%)	49 (70.0)	29 (69.0)	
Female, n (%)	21 (30.0)	13 (31.0)	.92^b^

a Student's *t* test.

b Chi‐square test.

Differences in RIPLS total score before and after TBL between uniprofessional groups and multiprofessional groups.

### 3.2 Univariate analysis

**Figure 2:**
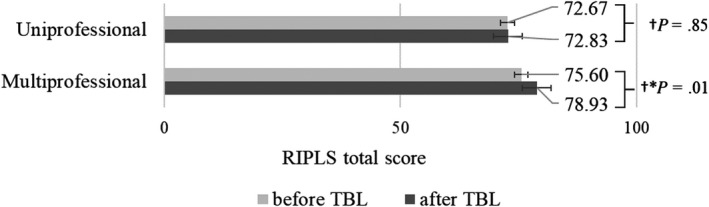
RIPLS total score before and after TBL in both groups. ^†^Paired *t* test, *P value <.05.

Table 2 shows the RIPLS total score and the mean difference in the RIPLS total score before and after the TBL program in the uni‐/multiprofessional groups. The RIPLS total score was 72.67 ± 6.84 (average ± standard deviation) points before the program and 72.83 ± 8.99 points after the program in the uniprofessional groups (mean difference of 0.16 points, *P* = .85 [paired *t* test]; 95% confidence interval [CI]: −1.46 to 1.77, effect size: 0.02) (Table 2, item (1); and Figure 2). The RIPLS total score was 75.60 ± 10.24 points before the program and 78.93 ± 11.63 points after the program in the multiprofessional groups (mean difference of 3.33 points, *P* = .01 [paired *t* test]; 95% CI: 1.03 to 5.63, effect size: 0.42) (Table 2, item (2); and Figure 2). There was no statistically significant difference in RIPLS total score before the program between the uniprofessional and multiprofessional groups (difference of 2.93 points, *P* = .11 [Student's *t* test]; 95% CI: −0.63 to 6.48) (Table 2, item (3)). In contrast, the mean difference in RIPLS total score before and after the program was significantly higher in the multiprofessional groups than in the uniprofessional groups (3.33 points vs 0.16 points, difference of 3.17 points, *P* = .02; 95% CI: 0.47‐5.88) (Table 2, item (4)).

**Table 2:** Mean difference in RIPLS total score before and after the TBL program in the two groups

	Uniprofessional group (n = 70)	Multiprofessional group (n = 42)
Before the TBL program	A: 72.67 ± 6.84	B: 75.60 ± 10.24
After the TBL program	C: 72.83 ± 8.99	D: 78.93 ± 11.63
Mean difference in RIPLS total score (After‐Before)	C‐A: 0.16	D‐B: 3.33

Note

Value (A‐D): RIPLS total score ± standard deviation.

Univariate analysis: (1) A vs C: compared between before and after the TBL program in the uniprofessional group, paired *t* test. (2) B vs D: compared between before and after the TBL program in the multiprofessional group, paired *t* test. (3) A vs B: compared between the uni‐ and multiprofessional groups before the TBL program, Student's *t* test. (4) D‐B vs C‐A: mean difference in score between the uni‐ and multiprofessional groups, Student's *t* test.

### 3.3 Multiple regression analysis

Multiple regression analysis (forced entry method) was performed with adjustment for the RIPLS total score before the TBL program (dependent variable: mean difference in RIPLS total score before and after the TBL program; independent variables: RIPLS total score before the TBL program, group [multi‐/uniprofessional], age, and gender), and showed a significant standardized partial regression coefficient (*β* = 0.24; *P* = .01) for group (multi‐/uniprofessional). This suggests that after adjustment for the RIPLS total score before the program, the increase in RIPLS total score was larger in the multiprofessional groups than in the uniprofessional groups (Table 3).

**Table 3:** Multiple regression analysis with adjustment for the effect of RIPLS total score before the TBL program

Independent variable	*B*	β	*P* value	95% confidence interval
(Constant)	6.72		.45	−10.86 to 24.30
Multi‐/uniprofessional group	3.49	0.24	.01*	0.71 to 6.27
RIPLS total score before TBL	‐0.12	‐0.14	.14	−0.28 to 0.04
Gender	0.87	0.06	.55	−2.04 to 3.78
Age	0.06	0.02	.83	−0.49 to 0.61

Note

Dependent variable: difference between the RIPLS total score before the TBL program and that after the TBL program.

*B*: nonstandardized partial regression coefficient.

*β*: standard partial regression coefficient.

* *P* < .05.

## 4 DISCUSSION

We examined whether learning in a multiprofessional group improves the readiness of medical students for interprofessional learning compared to learning in a uniprofessional group composed of medical students only. There are three possible reasons for a potential difference.

The first is TBL. TBL is a learning strategy that involves the crucial interactions necessary for IPE. A previous study reported that the effect of these interactions was to improve medical students’ readiness for interprofessional learning.^24^ In a clinical practice framework, however, medical students who practiced in pairs with nursing students did not significantly improve their attitude toward IPE,^17^ suggesting that it may be important to choose learning strategies that intentionally incorporate interaction.

Second, at the time of the study, participants from both universities had just started studying subjects in their chosen specialty. A study reported that differences in knowledge may be an obstacle for equal interactions between professionals.^25^ This effect may be particularly pronounced for nonmedical undergraduate students, who may find it difficult to achieve equal interaction because they expect medical students to lead due to their perception of them as doctors.^26^ However, the medical students in our study had not started learning about clinical medicine, indicating that the level of knowledge between medical students and healthcare students was not markedly different. This may have resulted in a flat and smooth interaction between medical students and other students and improved readiness for interprofessional education. Although all medical students learned IPE based on the case scenario, there was no increase in RIPLS total score in the uniprofessional groups because they did not interact with students from other professions. Further, participants were lower‐year students who did not have concrete ideas about other professions, and thus had low readiness for IPE.^27^, ^28^

Third, it may be related to motivation by nonmedical undergraduate students toward interprofessional education and readiness status. Because the course was an elective subject for nursing, physical therapy, occupational therapy, and radiology students, there is a possibility that they had a high level of motivation for participation. Medical students in the multiprofessional groups may have been motivated by the active participation of nonmedical students and may have been influenced by students who were preparing to collaborate and be responsible for facilitation in the group. This may have led to the increased readiness among medical students toward IPE.

This study has three main strengths. First, randomized allocation was performed. Although random assignment is an important method for increasing the accuracy of the measured educational effect, it is often difficult to perform in the undergraduate educational setting, and the number of studies using random assignment is accordingly limited^29^. We were able to compare readiness for IPE between uniprofessional groups containing only medical students and multiprofessional groups containing medical and other healthcare students because we introduced a compulsory IPE program at X University with the cooperation of students from Y University who wanted to participate in the IPE program. Our findings are meaningful because we were able to use a research design that solved a number of problems that had arisen in prior research.

Second, we focused on medical students. We showed that IPE improved the readiness of medical students for interprofessional learning, even among medical students who were considered to possess a low level of readiness for interprofessional learning.^3^, ^22^, ^30^

Third, while many reports have described interprofessional learning programs in Europe and the United States, there are few such reports from Asia.^13^, ^29^, ^31^ Given that the most suitable type of IPE learning strategy in Asian countries has yet to be established, our finding that an interprofessional learning program using a TBL learning strategy was effective in Japan provides an important foundation for further research.

The results of unpaired t test analysis of the mean difference in scores before and after TBL in both groups for each of the 19 items of RIPLS are shown in Table S1. We found that there was a significant difference in items 3, 10, 11, and 12 before Bonferroni correction. While these individual significant differences may be potentially meaningful to our result, there were no significant differences after Bonferroni correction (significance level *P* = .05/19 = 0.0026). In most previous studies, RIPLS scores were evaluated for the whole questionnaire or for subscales.^16^, ^31^ Given that no studies have reported significant differences in individual items of the RIPLS, we examined the RIPLS total score.

Several limitations of this research warrant mention. First, RIPLS was used as a tool to evaluate readiness for interprofessional collaboration. However, some reports have raised doubts about the reliability and validity of RIPLS. Mahler et al^32^ suggested that verification using RIPLS was inadequate because there was a problem with evaluating attitudes toward IPE. Schmitz and Brandt suggested that it was inevitable that measurements would be close because faculty members taught many similar elements such as communication, cooperativeness, collaboration, understanding of roles and responsibilities, and teamwork at the same time in IPE, and that these had high internal correlations.^33^ However, a complete tool to measure the educational effect on interprofessional learning is not available. For evaluations using the whole RIPLS, internal validity has been verified in many studies, and the results currently suggest that the whole RIPLS can be used as one evaluation scale. Second, there may have been information bias. Compared to the medical students who were randomized into the uniprofessional groups, those in the multiprofessional groups may have been more likely to overreport their readiness for interprofessional learning after the IPE program because they were not blinded to group assignment and may have felt that they were expected to report increased readiness. Alternatively, the medical students who were assigned to the multiprofessional groups may naturally have taken an interest in IPE and some may have studied IPE in advance. Therefore, the increase in RIPLS total score after the TBL program may have been due not only to the TBL class but also to knowledge of the grouping itself. Similar to another education study,^34^ it is difficult to “blind” learners to an assigned group in education studies. Third, participants were limited to students from two universities and the sample size was small; therefore, generalizability may be limited. Further investigation is necessary. Fourth, we cannot rule out the possibility that the ratio of students of each profession may have affected the results of IPE. To our knowledge, there is no prior study on the optimal ratio of students of each profession. Although our research suggests that learning with multiple professions may improve medical students’ readiness for interprofessional learning, it does not allow us to determine the optimal student ratio. This should be determined in future studies.

In the IPE program using the TBL format, which was conducted to improve readiness for interprofessional collaboration, we found that medical students who learned through participation in a multiprofessional group had improved readiness for interprofessional learning. This improvement in readiness may have been due to the fact that the participating medical and healthcare professional students were lower‐year students and that students of various healthcare professions were involved. Future studies should consider evaluation using other tools, a larger sample size, a greater number of institutions, and upper‐year students.

## 5 CONCLUSION

Our IPE program using a TBL format conducted among second‐year medical students improved readiness for interprofessional learning among medical students assigned to multiprofessional groups compared to those assigned to uniprofessional groups. IPE using TBL may be helpful for improving readiness for future IPW.

## CONFLICT OF INTERESTS

The authors have stated explicitly that there are no conflicts of interest in connection with this article.

## ETHICAL APPROVAL

This study was approved by the Ethics Committee of the University of Tsukuba (No. 793 in 2013).
